# Phytochemical Profiles and Anticancer Effects of *Calophyllum inophyllum* L. Extract Relating to Reactive Oxygen Species Modulation on Patient-Derived Cells from Breast and Lung Cancers

**DOI:** 10.1155/2023/6613670

**Published:** 2023-07-20

**Authors:** Jetsada Ruangsuriya, Jirapast Sichaem, Apichat Tantraworasin, Somcharoen Saeteng, Phanchaporn Wongmaneerung, Angkhana Inta, Neal M. Davies, Kewalin Inthanon

**Affiliations:** ^1^Department of Biochemistry, Faculty of Medicine, Chiang Mai University, Chiang Mai, Thailand; ^2^Functional Food Research Center for Well-Being, Science and Technology Research Institute, Chiang Mai University, Chiang Mai, Thailand; ^3^Research Unit in Natural Products Chemistry and Bioactivities, Faculty of Science and Technology, Thammasat University Lampang Campus, Lampang, Thailand; ^4^Clinical Surgical Research Center, Chiang Mai University, Chiang Mai, Thailand; ^5^Department of Surgery, Faculty of Medicine, Chiang Mai University, Chiang Mai, Thailand; ^6^Department of Biology, Faculty of Science, Chiang Mai University, Chiang Mai, Thailand; ^7^Faculty of Pharmacy & Pharmaceutical Sciences, University of Alberta, Edmonton, Alberta, Canada; ^8^Department of Biotechnology, Faculty of Science and Technology, Thammasat University Lampang Campus, Lampang, Thailand

## Abstract

Reactive oxygen species (ROS) contribute to cancer growth and metastasis. Using antioxidants to modulate cellular ROS levels is a promisingstrategy for cancer prevention and treatment. *Calophyllum inophyllum* L., or tamanu, is a medicinal plant renowned for its anti-inflammatory, antioxidant, and anticancer properties in traditional medicine systems. However, the anticancer effects of *C. inophyllum* extract on cellular ROS remain unexplored. This study represents the first report on such effects and provides the potential mechanisms underlying the anticancer properties of *C. inophyllum* extract. The branches of *C. inophyllum* were extracted, and the extract was comprehensively analyzed for phytochemical constituents, antioxidant capacity, total phenolic content, and total flavonoid content. Subsequently, the extract's potential anticancer properties were evaluated using patient-derived cells from breast and lung cancer. The results revealed that the *C. inophyllum* extract possesses notable antioxidant activity and demonstrated no cytotoxicity within the initial 24 h of treatment. However, after 72 h, it exhibited significant antiproliferative effects. Moreover, the extract exhibited inhibitory properties against migration and invasion at concentrations below the IC_50_, which corresponded to the expression of related genes. Notably, these effects correlated with the reduction of intracellular ROS levels. Overall, our findings highlight the anticancer potential of *C. inophyllum* extract, emphasize its ability to modulate cellular ROS levels and target key molecular pathways involved in cancer progression. This study sheds light on the promising therapeutic implications of *C. inophyllum* extract as a novel agent for cancer treatment, which is safe for normal cells.

## 1. Introduction

Reactive oxygen species (ROS) play many crucial roles in cancer progression and metastasis leading to the death of cancerous patients. As we have known for many decades that lung cancer in males and breast cancer in females are the leading causes of mortality worldwide [1]. ROS is accused of cancer death due to the involvement of cancer progression and metastasis including cell growth, proliferation, migration, and invasion by activating upstream-proliferative signaling cascades, growth factor receptors, adhesion molecules, and transcription factors [2]. Nevertheless, disrupting redox homeostasis in cancer cells by rapidly increasing intracellular ROS ultimately results in cell death which is a property of several FDA-approved drugs for cancer treatment including paclitaxel, 5-fluorouracil, doxorubicin, and cisplatin [3]. These effective chemotherapies also result in serious side effects on surrounding normal cells by oxidative damage. Increasing the ROS level above the redox balance in cancer cells effectively kills the cells while the ROS level below that alleviates aggressiveness by deceleration of proliferation, metastasis, and cell death induction [4]. Reduction of intracellular ROS by antioxidants on ROS-dependent cancer cells enhanced the potential of success in the treatment of metastatic solid tumors demonstrated in either *in vitro* or *in vivo* models with promising results [5, 6]. In addition, numerous plant-derived phytochemicals, including polyphenols and flavonoids, exhibit the potential for antiproliferation and antimetastasis against cancer cells without causing cytotoxicity [5–7].

*Calophyllum inophyllum* L., commonly known as tamanu, is a tropical evergreen tree belonging to the family Calophyllaceae. It distributes across Thailand, especially in the coastal regions. With its medium-sized stature, reaching heights of 20–30 meters, it thrives in diverse landscapes, including forests, mangroves, coastal areas, and tropical regions [8, 9]. This plant has been recognized as one of the most important medicinal plants in Ayurvedic and Thai traditional medicine with various parts including leaves, flowers, and stem barks with medicinal properties [10]. The plant extract from various parts is rich in antioxidants identified as polyphenols, phenolic acids, flavonoids, and other antioxidant structuring phytochemicals which exhibit anti-inflammation, antimicrobial, and anticancer effects [8, 11, 12]. The phytochemicals obtained from the ethanolic leaf extract and oil seed of this plant have demonstrated cytotoxic effects on breast and lung cancer cells, leading to the induction of apoptosis [13, 14]. In addition, the extract from this plant was noncytotoxic to many noncancerous cells including keratinocytes, dermal fibroblasts [15], preosteoblasts [16], and conjunctival epithelial cells [17].

Despite its traditional use and documented properties, there is a significant knowledge gap in the scientific literature regarding the specific effects of *C. inophyllum* on cancer treatment and its relationship with intracellular ROS. Further research is needed to explore the potential anticancer effects of *C. inophyllum* extract and its modulation of ROS. With its potent antioxidant, anti-inflammatory, and anticancer properties, *C. inophyllum * presents an intriguing candidate for investigating cellular ROS modulation in cancer cells. Its ability to impact multiple pathways involved in cancer development makes *C. inophyllum* a promising subject for scientific inquiry.

The objectives of this study were to investigate the phytochemical properties of *C. inophyllum* extract and to elucidate the effects of the *C. inophyllum* extract on cancer cells derived from the patients in comparison with the cancer cell lines. While previous literature has extensively explored the therapeutic potential of various parts of *C. inophyllum*, the branches of the tree have received limited attention until now. The study assessed the biochemical properties of the *C. inophyllum* branch extract and identified its phytochemical constituents. It was hypothesized that using cancer cells derived from the patients might provide better outcomes due to the fact that they demonstrate characteristics of cancer tissues in the body which promptly respond to the extract.

## 2. Materials and Methods

### 2.1. Plant Materials

The fresh branches of *C. inophyllum* were collected from Chomphu Subdistrict, Mueang District, Lampang Province, Thailand, in February 2022. The plant was identified and authenticated by a taxonomist, Dr. Angkhana Inta, Department of Biology, Faculty of Science, Chiang Mai University, Thailand, and deposited at the Queen Sirikit Botanic Garden Herbarium (QBG), Thailand (voucher specimen no. 7885). The plant branches were stripped of all leaves, cut into small crumbs, air-dried in a hot air oven at 45°C for 7 days, and powdered using an electric blender. The obtained powder was stored in a light-protective glass bottle before further extraction procedures.

### 2.2. Extraction of Plant Materials

The ethanolic extract was prepared by macerating the 100 g dried powder with 70% ethanol for 72 h. The extracts were filtered using Whatman No. 1 filter paper (Whatman® Schleicher & Schuell, UK) and concentrated under reduced pressure at 45°C in a rotary evaporator (Buchi, Switzerland). Finally, the *C. inophyllum* extract was collected, weighed, calculated for its yield, and kept in a light-protected vial at 4°C for further experiments. For use, the extract was dissolved in the initial 70% ethanol.

### 2.3. Evaluation of Phytochemical Properties

#### 2.3.1. DPPH Free Radical Scavenging Assay

The antioxidant activity of *C. inophyllum* extract was estimated *in vitro* based on the scavenging activity of the 2,2-diphenyl-1-picrylhydrazyl (DPPH) free radical, as described by Blois [18]. The stock solution of the extract was diluted in methanol and pipetted into each well of a 96-well plate. Then, the DPPH solution was added to each well and thoroughly mixed with the plate shaker. The mixture was further incubated in the dark at room temperature for 30 min. The optical density values at 517 nm were measured by using a microplate reader (Rayto, China). The percentage of DPPH free radical scavenging activity was calculated. The half-maximal inhibitory concentration (IC_50_) was calculated from the plotted equation. Ascorbic acid and quercetin were used as standard antioxidants and positive controls.

#### 2.3.2. Total Phenolic Content

The total phenolic content of *C. inophyllum* extract was estimated by colorimetric assay, Folin–Ciocalteu's reagent as described elsewhere [19]. In brief, 40 *μ*l of the sample was pipetted into glass tubes, 800 *μ*l 10% Folin–Ciocalteu's reagent was topped up, and the mixture was well mixed and incubated at room temperature for 5 min. Then, 800 *μ*l 7% sodium carbonate and 360 *μ*l ultrapure water were added to each tube of the mixture followed by thorough mixing. The reaction mixture was incubated for 2 h in the dark at room temperature. The OD_760_ was measured using a UV-VIS spectrophotometer (Hitachi, Japan). The OD_760_ values of quercetin were plotted to obtain the calibration curve. Estimation of total phenolic content in *C. inophyllum* extract is exhibited as mg of gallic acid equivalent per gram dry extract (mg GAE/g DW) using the calibration curve of gallic acid.

#### 2.3.3. Total Flavonoid Content

Total flavonoid content of the *C. inophyllum* extract was assessed by the aluminum chloride colorimetric assay as described previously [20]. 0.2 ml of either *C. inophyllum* extract or standard was mixed with 4.8 ml of ultrapure water in a test tube. 0.3 ml of 5% NaNO_2_ was topped up and mixed well by using a vortex mixer. After that, the mixture was left at room temperature for 5 min prior to the addition of 0.3 ml of 10% AlCl_3_ 6H_2_O as well as 2 ml of 1 M NaOH solution into each tube. The ultrapure water was dispensed to reach the final volume of 10 ml. The optical density values at 415 nm were recorded using a UV-VIS spectrophotometer (Hitachi, Japan). Quercetin was used as a standard. The total flavonoid content was expressed as mg of quercetin equivalents per gram of dry extract (mg QE/g DW) using the calibration curve of quercetin.

#### 2.3.4. Gas Chromatography-Mass Spectrophotometry Analysis

In order to identify certain phytochemical constituents as well as to illustrate the chromatogram fingerprint in the *C. inophyllum* extract, GC-MS analysis was performed using Agilent Technologies GC-MS 7890A/5975C Series (Agilent, USA) equipped with a capillary column (30 m in length × 0.25 mm in diameter × 0.25 *μ*m film thickness) (partly modified from Kadir [21]). The spectrum database reported in the W08N08 library (John Wiley & Sons, Inc., USA) was used to identify the phytochemical components of the extract. The identified phytochemicals were examined using PubChem (https://pubchem.ncbi.nlm.nih.gov/).

### 2.4. Cancer Tissue Collection, Establishment, and Culture of Patient-Derived Cells

Biological waste cancer tissues were collected from 6 anonymous patients after surgery by physicians. All processes were approved by the Research Ethics Committee, Faculty of Medicine Chiang Mai University (EXEMPTION 8600/2021). The samples were stored in a transport medium (Dulbecco's modified Eagle medium (DMEM) + 5% fetal bovine serum (FBS) + 1X antibiotics-antimycotics) on ice and subjected for cell outgrowth preparation. After rinsing with sterile phosphate buffer saline (PBS) containing 1X antibiotics-antimycotics, the tissues were minced plated onto a culture flask (Nunc™; Thermo Fisher Scientific, USA) filled with complete medium (20% FBS in DMEM+1X antibiotics-antimycotics). The flasks were incubated in a CO_2_ incubator at 37°C till the outgrowth cells reached 50% confluence. Then, subculture was performed and continued maintaining the cells with DMEM supplemented with 1X antibiotics-antimycotics and 10% FBS. These cells were called patient-derived cells (PDCs), which were derived from breast and lung cancers, abbreviated as BC and LC, respectively.

### 2.5. Ki-67 Immunocytochemistry for Cancer Cell Characterization

This experiment aimed to determine whether the PDCs possess characteristics of cancer cells by assessing their proliferation capability using the cancer proliferation marker, Ki-67. The expression of Ki-67 was examined in the PDCs and compared to that of standard cancer cell lines, serving as a reference. After the 24-hour culture, the cells were fixed with an ice-cold 4% paraformaldehyde (Sigma, USA) for 15 min. The fixed cells were rinsed with PBS and permeabilized with 0.25% Triton-X at room temperature for 20 min. The permeabilized cells were washed with PBS prior to submerging them into a blocking solution containing 10% bovine serum albumin (Sigma, USA) at room temperature for 30 min. Primary antibody of Ki-67 (1 : 5,000) (Thermo Fisher Scientific, USA) was applied to the cell overnight at 4°C. After a thorough wash with PBS, the diluted peroxidase-conjugated secondary antibody (1 : 2,000) (Elabscience, USA) was applied, and DAB substrate (3,3′-diaminobenzidine tetrahydrochloride) (Elabscience, USA) was added. The cells were then counterstained with hematoxylin (Sigma, USA). The stained cells were photographed under a microscope, and the number of positive nuclei was counted and used to calculate the mean percentage of positive cells.

### 2.6. Cell Lines and Culture Conditions

Two lines of human lung carcinoma cells (A549 and NCI-H1299), two lines of breast carcinoma cells (MCF-7 and MDA-MB-231), and a noncancerous normal human lung fibroblast (IMR-90) were used in this research. All cell lines were purchased from American Type Culture Collection (USA). A549 and IMR-90 were cultured and propagated in DMEM, whilst NCI-H1299 was in RPMI 1640. The complete medium was supplemented with 100 U/ml penicillin, 100 *μ*g/ml streptomycin, and 10% FBS. All cells were maintained under a standard cell culture condition at 37°C in an atmosphere consisting of 5% CO_2_.

### 2.7. Evaluation of *C. inophyllum* Extract on Cancer Bioactivities

#### 2.7.1. Effects on Cytotoxic and Antiproliferation

To determine whether *C. inophyllum* extract had cytotoxicity and antiproliferative effects on cancer cells, a sulforhodamine B colorimetric assay was applied. After seeding cells into each well of a 96-well culture plate and incubating for 24 h, the cells were exposed to the complete medium containing various concentrations of *C. inophyllum* extracts for 24 and 72 h, along with quercetin. The assessment of cell viability was performed using the sulforhodamine B colorimetric assay, as described by Vichai and Kirtikara [22]. In brief, cells were fixed with 10% trichloroacetic acid and incubated with 0.057% sulforhodamine B solution (Sigma, USA) at room temperature for 30 min. After washing with 1% acetic acid (Sigma, USA), the dye was solubilized with 10 mM tris solution (pH 10.5) (Sigma, USA) with constant agitation for 15 min. The optical density values at 510 nm were read by a microplate reader (Rayto, China) and the IC_50_ of the extract was determined by using PriProbit Program ver. 1.63 [23]. The criteria for cytotoxicity were adhered to when the IC_50_ value was higher than 20 *μ*g/ml [24]. In addition, selectivity index values were calculated by dividing the IC_50_ values of the normal cell line by the IC_50_ values of the cancer cells. A selectivity index higher than 1.00 indicates specificity towards cancer cells [25]. The IMR-90 cell line was used as the normal cell line in this experiment.

#### 2.7.2. Effects on Cell Migration

To assess the inhibitory effects of the *C. inophyllum* extract on cancer cell migration, a migration assay was employed. After the cells reached 75% confluence and formed a monolayer, the cell layers were scratched with a sterile pipette tip and washed with PBS. The complete medium containing *C. inophyllum* extract and reference compound was then added to the scratched cell layer prior to the incubation under the standard culture condition. Gap closure was monitored and photomicrographed after 24 h using an Optika IM-3 inverted microscope equipped with a C-B10+ digital camera (Optika, Italy) in comparison with 0 h of incubation. The gap distances were measured using ImageJ software (NIH, USA), and the results were expressed as percentages of the gap distance in the treatment group relative to the gap distance in the untreated control at 0 h.

#### 2.7.3. Effects on Cell Invasion

To investigate the potential inhibitory effects of *C. inophyllum* extract on cancer cell invasion, a Transwell insert coated with Matrigel® was utilized. The invasion assay was performed in 24-well Corning® Biocoat® Matrigel® invasion chambers (Corning, USA) with a membrane pore size of 8 *μ*m. According to the manufacturer's instructions, the chambers were rehydrated and prewarmed by adding 37°C PBS in each chamber containing the Matrigel® insert (upper chamber) and incubated at 37°C for 2 h. Cells were plated onto the insert supplemented with the complete medium containing *C. inophyllum* extract or a reference substance. The lower chamber was filled with a medium containing 20% FBS as a chemoattractant. After 24 h, the inserts were fixed in 4% paraformaldehyde and stained with 0.1% crystal violet solution (Sigma, USA). The invaded cells on the lower surface of the inserts were photographed and counted. The complete medium supplemented with 10% FBS was used as a negative control. The results were quantified as the percentage of invaded cells, calculated by multiplying the ratio of cell numbers in the treatment group to the cell numbers in the negative control by 100.

#### 2.7.4. Effects on Intracellular Reactive Oxygen Species (ROS) Modulation

To determine intracellular levels of ROS in the form of hydrogen peroxide (H_2_O_2_) and superoxide anions (O_2_·^−^), 2,7-dichlorodihydrofluorescein diacetate (DCFDA) was applied. After seeding the cells and incubating them in standard culture conditions for 24 h, the cells were exposed to the complete medium containing either the *C. inophyllum* extract, the reference compound, or 100 *μ*M *tert*-butyl hydroperoxide (TBHP, a ROS-inducing standard compound) [26]. Intracellular ROS determination was conducted after 24 h of exposure to the targeted substances using the DCFDA/H2DCFDA—Cellular ROS Assay Kit (Abcam, UK). According to the manufacturer's instructions, the medium was discarded, and the cells were rinsed with PBS and with 1X reaction buffer. 1X DCFDA solution was dropped onto the cell surface and incubated at 37°C for 45 min in the dark. The cells were rinsed with PBS prior to the observation under a fluorescence microscope (Olympus, Japan). Fluorescence intensity was measured by using ImageJ software (NIH, USA) through digital photomicrographs. The data were represented as the percentages of fluorescence intensity in comparison to the negative control, untreated cells.

#### 2.7.5. Effects on Gene Expression

To confirm the effects of *C. inophyllum* extract on the inhibition of cancer cell migration, invasion, and ROS modulation, quantitative real-time PCR was used to quantify the expression levels of certain genes involved in the abovementioned processes. mRNA from the cells exposed to the *C. inophyllum* extract or quercetin for 24 h was harvested using NucleoSpin® RNA/Protein isolation (Macherey-Nagel™, Germany). Total cDNA was subsequently synthesized by using ReverTra Ace™ qPCR RT Master Mix (Toyobo, Japan). The expression of the candidate genes involved in cell migration (*E-cadherin* and *Twist-1)*, invasion (*MMP-2* and *MMP-9*), and ROS responsive (*NRF2* and *HIF-1α*) were evaluated. The primer sequences of the gene were as follows: *E-cadherin* (forward primer 5′- AGCGAGTGGATGCCGCCTTTAA -3′ and reverse primer 5′- CATTCCAGGCATCTGCGATGAG -3′), *Twist-1* (forward primer 5′- GCCAGGTACATCGACTTCCTCT -3′ and reverse primer 5′- TCCATCCTCCAGACCGAGAAGG -3′) [27], *MMP-2* (forward primer 5′- AGCGAGTGGATGCCGCCTTTAA -3′ and reverse primer 5′- CATTCCAGGCATCTGCGATGAG -3′), *MMP-9* (forward primer 5′- GCCACTACTGTGCCTTTGAGTC -3′ and reverse primer 5′- CCCTCAGAGAATCGCCAGTACT -3′) [28], *NRF2* (forward primer 5′- CACATCCAGTCAGAAACCAGTGG -3′ and reverse primer 5′- GGAATGTCTGCGCCAAAAGCTG -3′) [27], *HIF-1α* (forward primer 5′- TATGAGCCAGAAGAACTTTTAGGC -3′ and reverse primer 5′- CACCTCTTTTGGCAAGCATCCTG -3′) [29], and *β-actin* (forward primer 5′- CACCATTGGCAATGAGCGGTTC -3′, and reverse primer 5′- AGGTCTTTGCGGATGTCCACGT -3′) [28]. Quantitative real-time PCR was performed in Bioer Real Time PCR Linegene K Plus (Bioer, China) using Thunderbird® SYBR® qPCR Mix (Toyobo, Japan). *β-actin* was applied as a housekeeping gene. Untreated cells were used as a control. Fold changes of gene expression were calculated by the 2^−ΔΔCt^ method [30].

### 2.8. Statistical Analysis

The data presented in this study are expressed as the mean ± standard deviation (SD) and were derived from five independent replicates of each treatment. The significant differences among groups were evaluated by ANOVA followed by the Tukey *post hoc* test with a confidence interval *p* < 0.01.

## 3. Results

### 3.1. Extraction of Plant Materials

The extraction yield was approximately 9.06% viscous mass obtained from 100 g powder branches of *C. inophyllum* after 72 h of continuous extraction in ethanol.

### 3.2. Evaluation of Phytochemical Properties

#### 3.2.1. DPPH Free Radical Scavenging Activity

The antioxidant activity of *C. inophyllum* extract was investigated by a DPPH free radical scavenging method. DPPH scavenging activity at IC_50_ of *C. inophyllum* extract was determined in comparison to ascorbic acid and quercetin by the linear equation method and the IC_50_ values of the *C. inophyllum* extract were the highest compared to ascorbic acid and quercetin (Table 1).

#### 3.2.2. Total Phenolic and Flavonoid Contents

The total phenolic content and the total flavonoid content of the *C. inophyllum* extract were determined as 109.16 ± 1.21 mg GAE/g DW and 96.88 ± 0.89 mg QE/g DW, respectively.

#### 3.2.3. GC-MS Analysis of *C. inophyllum* Extract

**Figure 1:**
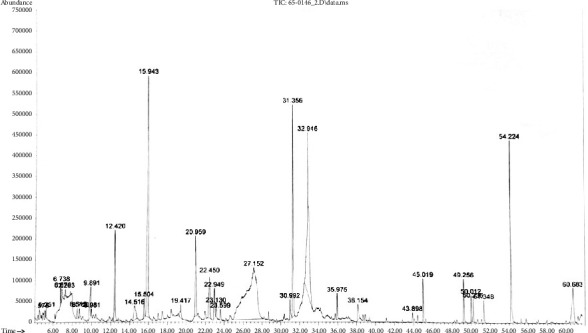
The GC-MS chromatogram of the *C. inophyllum* extract.

The GC-MS profile of the ethanolic extract of *C. inophyllum* is shown in Figure 1. The main constituents identified in the extract are reported in Table 2. Sixteen phytochemicals were identified (the quality index >80%). Most components were phenolic (approximately 31% of total identified compounds). Other components were classified as furoic acid esters, dihydropyranones, catechols, benzofurans, furans, fatty acids, fatty acid esters, isoquinolines, and xanthones. The three highest abundant phytochemicals found in the *C. inophyllum* extract were 5-hydroxymethylfurfural (5-HMF), antiarol, and syringol which were classified as furans, phenols, and phenols, respectively.

### 3.3. Cancer Tissue Collection, Establishment, and Culture of Patient-Derived Cells

**Figure 2:**
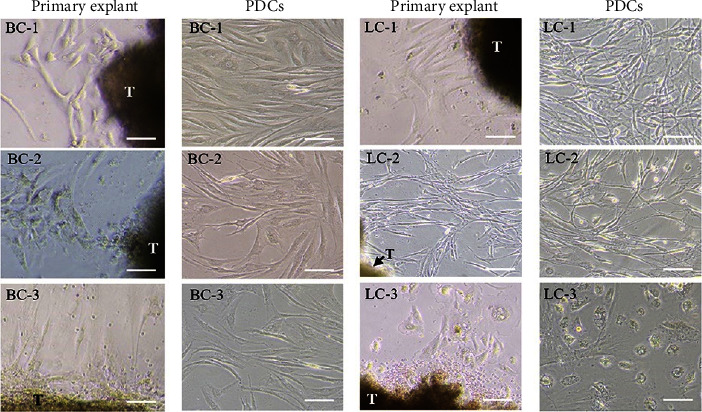
Cancer cell outgrowth from cancer tissue (primary explant) and PDCs of breast (BC-1, BC-2, and BC-3) and lung (LC-1, LC-2, and LC-3) cancers. PDCs = patient-derived cells, T = cancer sample tissue, and scale bar = 150 *μ*m.

Cancer biopsies were collected from patients (Table 3) and disaggregated to primary cancer cells. Cells were outgrown from the cancer tissue samples and subcultured to obtain PDCs of breast (BC-1, BC-2, and BC-3) and lung (LC-1, LC-2, and LC-3) cancer (Figure 2). All breast and lung cancer samples were diagnosed by the pathologists as invasive carcinoma and adenocarcinoma, respectively. Phenotypic heterogeneity of the populations was found in all BC and LC cells. Fibroblast-like cells were the dominant phenotype in most of the cells in the population, while mesenchymal-like and epithelial-like cells were in the minority.

### 3.4. Ki-67 Immunocytochemistry for Cancer Cell Characterization

**Figure 3:**
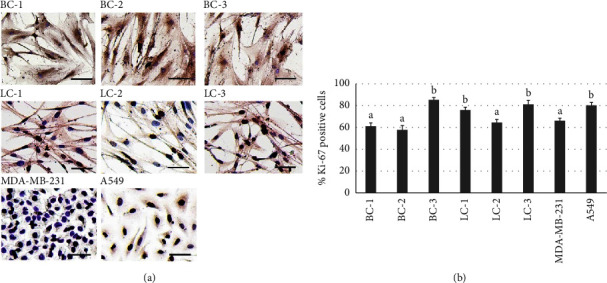
Expression of Ki-67 in PDCs and cancer cell line by immunostaining: (a) areas with Ki-67-positive cells expressed brown nuclei. Scale bar = 200 *μ*m. (b) Histogram indicating the percentage of Ki-67-positive cells in patient-derived cells (PDCs) of breast (BC-1, BC-2, and BC-3) and lung (LC-1, LC-2, and LC-3) cancers and in standard cancer cell lines of breast (MDA-MB-231) and lung (A549) cancers. Significant differences are indicated by different alphabets above the bars (a, b) at *p* *<* 0.01.

Immunocytochemistry of Ki-67 was applied to BC and LC cells in comparison to A549 and MDA-MB-231. The positive cells expressed brown nuclei (Figure 3(a)) were randomly counted and calculated to the percentage of positive cells (Figure 3(b)). There were 2 groups categorized by the percentage of positive cells; (1) >70%; BC-3, LC-3, LC-1, and A549 and (2) >50%; BC-2, BC-1, LC-2, and MDA-MB-231 (*p* *<* 0.01). PDCs exhibited characteristics of cancer proliferation identical to cancer cell line, A549, and MDA-MB-231.

### 3.5. Evaluation of *C. inophyllum* Extract on Cancer Bioactivities

#### 3.5.1. Effects on Cytotoxicity and Antiproliferation

The cytotoxicity and antiproliferative effects of the *C. inophyllum* extract were assessed by determining the IC_50_ values after exposing the cells to different concentrations of the extract for 24 and 72 h (Table 4). Quercetin, an antioxidant flavonoid, was applied as a positive control. At 24 h, the IC_50_ values from most of the *C. inophyllum* extract treatments were >1,000 *μ*g/ml, except MCF-7. In contrast, the IC_50_ values of *C. inophyllum* extract at 72 h were <1,000 *μ*g/ml in most of the cells, except the PDCs of breast cancer with IC_50_ values >1,000 *μ*g/ml. Unlike *C. inophyllum* extract, IC_50_ values of quercetin treatments at 24 h were >1,000 *μ*g/ml in all cancer cell lines while IC_50_ of PDCs and IMR-90 were <500 *μ*g/ml. However, IC_50_ values at 72 h of quercetin against most cancer cells were more evident with values <200 *μ*g/ml, except in A549 with a value >1,000 *μ*g/ml.

Selectivity index of the *C. inophyllum* extract and quercetin was elucidated using the IC_50_ of the normal cell (IMR-90) and the IC_50_ of each treatment at each time point (Table 5). The acceptable SI value was >1.00 indicating the specificity of the compounds to cancer cells. The results revealed that both *C. inophyllum* extract and quercetin exhibited specificity towards most cancer cell types, not all cell types were affected by these substances.

Based on the obtained results, subsequent experiments were conducted using *C. inophyllum* extract at concentrations of 100 and 200 *μ*g/ml, as well as quercetin at concentrations of 10 and 20 *μ*g/ml. These concentrations were selected based on their demonstrated antioxidant capacity and nontoxicity to cells, as evidenced by the cell survival rate of 80% after 24 h of treatment.

#### 3.5.2. Effects on Cell Migration

**Figure 4:**
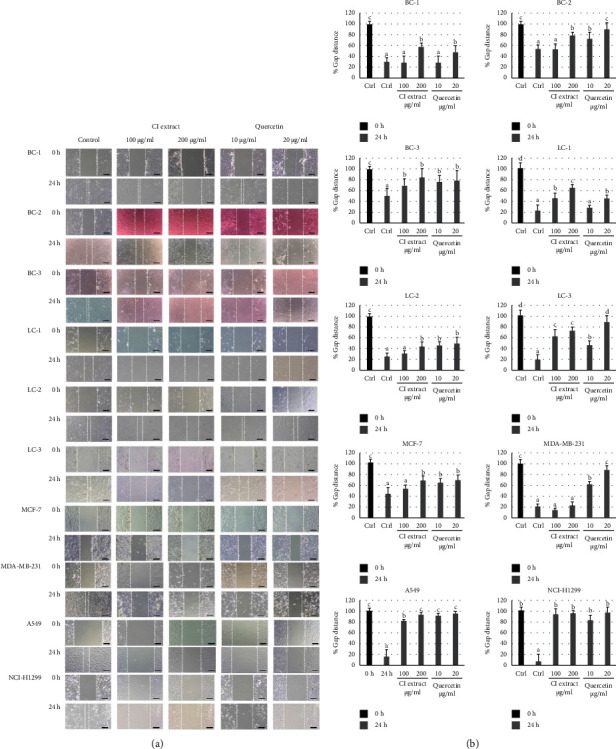
Effects of *C. inophyllum* extract on cancer cell migration. The inhibitory effects on cell migration of *C. inophyllum* extract (CI extract) at 100 and 200 *μ*g/ml and quercetin (quercetin) at 10 and 20 *μ*g/ml, which were noncytotoxic doses, were observed in patient-derived cells (PDCs) of breast cancer (BC-1, BC-2, and BC-3) and lung cancer (LC-1, LC-2, and LC-3), as well as in cancer cell lines of breast cancer (MCF-7 and MDA-MB-231) and lung cancer (A549 and NCI-H1299) after 0 h and 24 h treatments: (a) in the migration assay, the gap distances between the cell margins of the gaps (or between the white-dotted lines) were measured. Scale bar = 200 *μ*m. (b) Percentage of gap distance of the cells after 24 h of exposure to *C. inophyllum* extract and quercetin in comparison with the untreated control at 0 h (Ctrl, represented by darker column). Significant differences are indicated by different alphabets above the bars (a–d) at *p* *<* 0.01.

After treating the cells with *C. inophyllum* extract and quercetin in the migration assay, the gap distances were measured to assess their migratory ability. The results showed that both the *C. inophyllum* extract and quercetin treatments significantly closed the gap compared to the control group at 0 and 24 h (Figure 4(a)). In the photomicrographs, the white-dotted lines are used as guidelines to measure the gap distances between cell margins. The highest percentage of gap distance indicates the largest space observed between cell margins at 0 h (Figure 4(b)). After 24 h, the control group (Ctrl) displayed a significant decrease in the percentage of gap distance for all cells, suggesting cell migration and gap closure. However, the effects of *C. inophyllum* extract and quercetin treatments on gap distances varied depending on the cell types. At 200 *μ*g/ml of *C. inophyllum* extract exhibited significant inhibition of cell migration in most cancer cell types, except for MDA-MB-231. However, the effects of the extract at 100 *μ*g/ml were less pronounced, whilst quercetin at a concentration of 20 *μ*g/ml demonstrated significant inhibition of cell migration in most cancer cell types. However, at a concentration of 10 *μ*g/ml, quercetin showed less inhibitory ability, particularly in BC-1 and LC-1 cells. When comparing the effects of *C. inophyllum* extract to quercetin, the percentage of gap distance in the quercetin-treated cells indicated a greater potential for inhibiting cancer cell migration compared to the *C. inophyllum* extract across all cancer cell types.

#### 3.5.3. Effects on Cell Invasion

**Figure 5:**
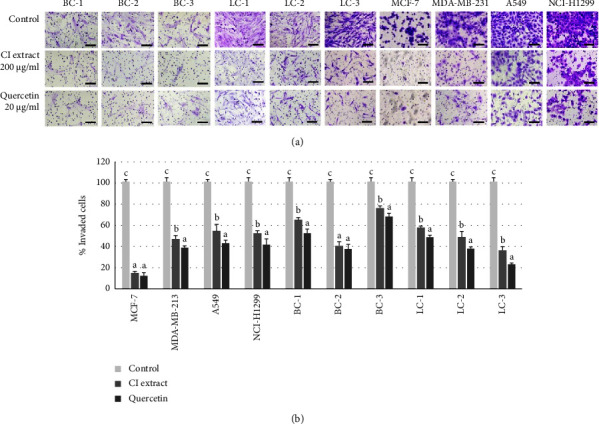
Effects of *C. inophyllum* extract on cancer cell invasion. The inhibitory effects of *C. inophyllum* extract at 200 *μ*g/ml (CI extract) and quercetin at 20 *μ*g/ml on cancer cell invasion were observed in patient-derived cells (PDCs) of breast (BC-1, BC-2, and BC-3) and lung (LC-1, LC-2, and LC-3) cancers, as well as in cancer cell lines of breast (MCF-7 and MDA-MB-231) and lung (A549 and NCI-H1299) cancers, after 24 h of treatment: (a) photomicrographs displayed the invading cells stained with crystal violet. Scale bar = 200 *μ*m. (b) Percentages of invaded cells after 24 h of exposure to *C. inophyllum* extract and quercetin in comparison to the control groups. Significant differences are indicated by different alphabets above the bars (a–c) at *p* *<* 0.01.

The most effective concentrations of *C. inophyllum* extract and quercetin were found to be 200 and 20 *μ*g/ml, respectively. Using these concentrations, the ability of the compounds to inhibit cancer cell invasion was tested using a Transwell insert with the Matrigel® model. Both the 200 *μ*g/ml *C. inophyllum* extract and 20 *μ*g/ml quercetin significantly inhibited cell invasion in all cancer cell types (Figure 5(a)). The results demonstrated a clear reduction in the number of invading cells compared to the controls for both the *C. inophyllum* extract and quercetin across all cancer cell types. Notably, quercetin treatments showed a greater potential to inhibit cancer cell invasion compared to the *C. inophyllum* extract, as observed by the percentage of invasion in the lower chamber (Figure 5(b)).

#### 3.5.4. Effects on Intracellular ROS Modulation

**Figure 6:**
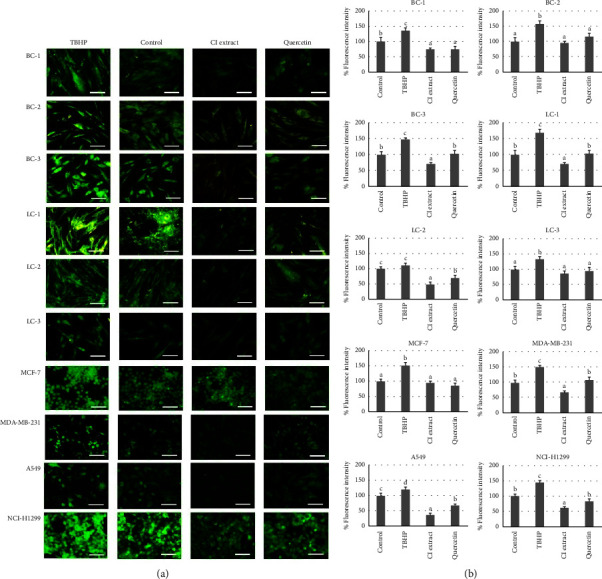
Effects of *C. inophyllum* extract on intracellular reactive oxygen species (ROS) modulation. The effects of 200 *μ*g/ml of *C. inophyllum* extract (CI extract) and 20 *μ*g/ml of quercetin (Quercetin) on ROS generation were evaluated in patient-derived cells (PDCs) from breast cancer (BC-1, BC-2, and BC-3) and lung cancer (LC-1, LC-2, and LC-3), as well as in breast cancer cell lines (MCF-7 and MDA-MB-231) and lung cancer cell lines (A549 and NCI-H1299) after 24 h of treatment. TBHP was used to activate ROS generation: (a) photomicrographs displayed intracellular ROS present in green fluorescence. Scale bar = 100 *μ*m. (b) Percentages of fluorescence intensity indicated the level of intracellular ROS after 24 h of exposure to *C. inophyllum* extract and quercetin in comparison to other treatments. Significant differences are indicated by different alphabets above the bars (a–c) at *p* *<* 0.01.

After exposing the cells to 200 *μ*g/ml of *C. inophyllum* extract or 20 *μ*g/ml of quercetin for 24 h, the intracellular ROS levels were visualized in green using fluorescence microscopy (Figure 6(a)). *tert*-Butyl hydroperoxide (TBHP), a compound known to induce ROS production, was used as a positive control and successfully increased ROS levels in all cancer cell types. The treatments with *C. inophyllum* extract and quercetin significantly reduced intracellular ROS levels compared to the untreated controls in all cancer cell types (Figure 6(b)). Notably, in many cases, the *C. inophyllum* extract exhibited a greater ability to decrease ROS levels compared to quercetin, except in MCF-7, BC-1, BC-2, and LC-3 cells where both *C. inophyllum* extract and quercetin treatments showed similar potency.

#### 3.5.5. Effects on Gene Expression

**Figure 7:**
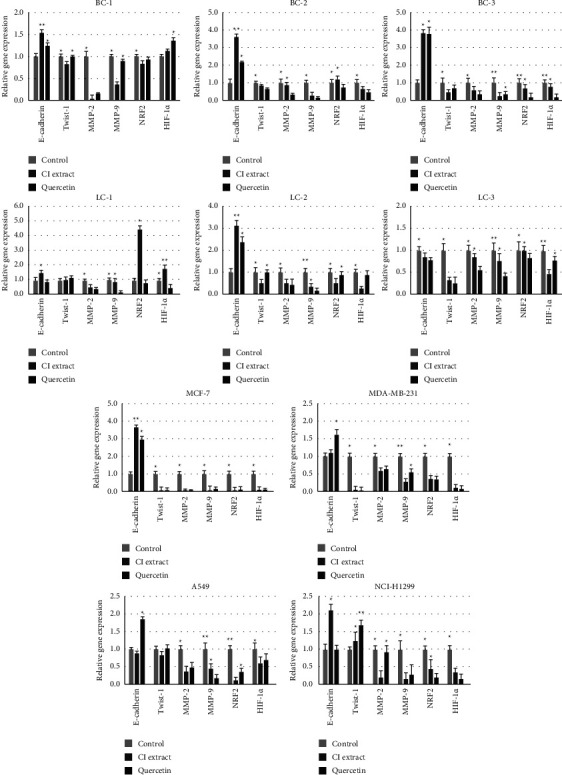
Effects of *C. inophyllum* extract on gene expression are presented as the relative gene expression of each treatment. The study examined the effects of 200 *μ*g/ml of *C. inophyllum* extract (CI extract) and 20 *μ*g/ml of quercetin (quercetin) on gene expression in patient-derived cells (PDCs) from breast cancer (BC-1, BC-2, and BC-3) and lung cancer (LC-1, LC-2, and LC-3), as well as in standard cancer cell lines from breast cancer (MCF-7 and MDA-MB-231) and lung cancer (A549 and NCI-H1299) after 24 h of treatment. The genes involved in migration, invasion, and ROS generation include *E-cadherin* and *Twist-1*, *MMP-2* and *MMP-9*, and *NRF2* and *HIF-1α*. The *Ct* values were collected and proceeded through 2^−ΔΔ*Ct*^ method in comparison to the housekeeping gene, *β-actin* as well as the untreated controls. The data were represented as mean ± S.D. Asterisks (^*∗*^ and ^*∗∗*^) on the top of the bar present significant differences in the relative expression of each gene among the treatments at *p* *<* 0.01.

The results demonstrated that treatment with 200 *μ*g/ml of *C. inophyllum* extract and 20 *μ*g/ml of quercetin significantly decreased the expression of genes involved in cancer cell migration, invasion, and ROS modulation, with the exception of *E-cadherin*, which showed significant activation (Figure 7). The expression of migration genes including *E-cadherin* and *Twist-1*, determined that *E-cadherin* was significantly activated in all cells treated, except LC-2. In contrast, the expression of *Twist-1* was significantly reduced, except in NCI-H1299. In addition, NCI-H1299 responded to either *C. inophyllum* extract or quercetin by significantly increasing the expression of *Twist-1*. Expressions of *MMP-2* and *MMP-9* as invasion-involved genes declined upon either *C. inophyllum* extract or quercetin treatments observed in all cancer cell types. However, a statistically insignificant reduction of those genes could be observed in certain cancer cell types including NCI-H1299, LC-1, LC-3, and BC-2. The ROS-responsive genes, *NRF2* and *HIF-1α* responded to the *C. inophyllum* extract and quercetin in a similar manner. The reductions of *NRF2* and *HIF-1α* were observed in all cell types, except LC-1 and BC-1. The *C. inophyllum*extract-exposed LC-1 enhanced the expression of *NRF2* and *HIF-1α*. In BC-1, the expression of *HIF-1α* from the treatments of *C. inophyllum* extract and quercetin was relatively higher than the controls; however, significant differences were found in the quercetin treatment.

## 4. Discussion

In this study, we investigated the effects of an ethanolic extract derived from branches of *C. inophyllum* on cancer cell viability, migration, and invasion. For the first time, we examined the extract's ability to alleviate intracellular ROS levels, an important factor in cancer progression. Our study was driven by the assumption that antioxidant extracts can effectively reduce free radicals in cells and potentially impede cancer progression.

We optimized the extraction conditions of *C. inophyllum* extract by using temperatures of 40–50°C and ethanol concentrations of 70–100% to preserve antioxidant properties and maximize bioactive compound yields [31]. The extract exhibited strong antioxidant activity (ranking from 100 to 150 *μ*g/ml) [32], demonstrated by its effective free radical scavenging capabilities (Table 1). GC-MS analysis confirmed the presence of phenolic compounds, flavonoids, and other bioactives, including the newly discovered 5-HMF, contributing to the extract's antioxidant activity (Table 2). These compounds, characterized by benzene rings with hydroxy or methoxy groups, possess direct and indirect antioxidant properties [33]. Previous studies consistently support the antioxidant capacity of *C. inophyllum*, including its stem bark and wood [34], highlighting its broad range of bioactivities and antioxidant potential [10]. Thus, *C. inophyllum* represents a valuable natural resource abundant in antioxidants and bioactive compounds. These compounds hold great promise for diverse therapeutic applications, including the development of anticancer therapies.

The PDCs offer distinct advantages over immortalized cell lines, as they faithfully preserve the cellular assembly, tissue architecture, microenvironment, and cancer niches found in the body. This unique characteristic enables PDCs to exhibit more accurate responses to cytotoxic compounds and enhanced detection of cancer markers, surpassing traditional cell lines [35]. A notable biomarker associated with cancer cell proliferation and metastasis is Ki-67, which is highly expressed in malignant breast and lung cancer cells but is minimally detected in normal proliferating cells [36]. In this study, the PDCs derived from breast or lung cancer demonstrated high Ki-67 expression (>50%) (Figure 3), a significant feature resembling that of cancer cells [37]. This further underscores the resemblance of these PDCs to malignant breast and lung cancer cells, emphasizing their utility as a valuable model for studying cancer biology.

Cytotoxicity and antiproliferation effects of *C. inophyllum* extracts were evaluated using both PDCs and standard cancer cell lines. A noncancerous cell line was included as a reference to assess the selective index. Quercetin, a natural flavonoid known for its high antioxidant activity, protective effects against oxidative damage, and anticarcinogenic properties, was used as a reference substance [38]. Our findings demonstrate that *C. inophyllum* extract and quercetin exhibited antiproliferative effects according to the NCI criteria [24] and consistent with previous studies [15–17], while showing no cytotoxicity (Table 4). The selectivity index indicated cancer specificity (>1.0) for most cells, with PDCs showing higher sensitivity to quercetin (Table 5). While the *C. inophyllum* extract lacked specificity for PDCs, it exhibited time-dependent antiproliferative effects. The presence of bioactive compounds, including 5-HMF and xanthones, likely contributed to the observed anticancer properties, either directly or indirectly [11, 12, 39]. Importantly, the extract demonstrated selectivity for cancer cells, making it a promising candidate for further anticancer drug development.

The antioxidant properties and cancer cell specificity of the *C. inophyllum* extract were found to be associated with its potential for inhibiting migration and invasion (Figures 4 and 5), which correlated with a reduction in intracellular ROS levels (Figure 6). This was further supported by the upregulation of *E-cadherin* and downregulation of *MMP-9*, *MMP-2*, *Twist-1*, *NRF2*, and *HIF-1α* (Figure 7). The extract and quercetin, known for their antioxidant capabilities, likely alleviated intracellular ROS, leading to the downregulation of *NRF2* and *HIF-1α*. Consequently, the expressions of *MMP-2* and *MMP-9* were partially restricted, inhibiting epithelial-mesenchymal transition (EMT) [40]. Previous studies have shown that reducing intracellular ROS can disrupt metastasis in various types of cancer by decreasing *MMP-2* and *MMP-9* expression [41, 42]. Similar effects have been observed with other antioxidants such as silibinin and quercetin derivatives [43, 44]. In addition, the downregulation of *NRF2* and *HIF-1α*, which are associated with cancer cell growth and propagation, has been reported in response to antioxidants [45]. The antioxidant phytochemicals in *C. inophyllum* extract, including 5-HMF, 2-methoxy-4-vinylphenol, cinnamic derivatives, xanthones, and isoquinolines, likely contribute to the anticancer effects by directly reducing intracellular ROS and inhibiting cell migration and invasion [11, 46].

However, our findings showed differential expression patterns of migration and invasion-related genes in LC-3 and NCI-H1299 cell lines treated with *C. inophyllum* extract and quercetin. Both treatments resulted in the downregulation of *E-cadherin* and *Twist-1* (Figure 7), suggesting their potential role in inhibiting metastasis. Notably, in highly metastatic lung cancer cells, the association between EMT and *MMPs* with metastasis appears to be mediated through integrin- and protease-independent mechanisms [47, 48]. Furthermore, 70% of cellular deformation under hypoxia was dependent on *Twist-1* and *MMPs* but not EMT process for their migration [49].

In addition, the exposure to *C. inophyllum* extract and quercetin led to the reduction of *NRF2* and *HIF-1α* in most cells, with BC-1 and LC-1 cells showing distinct responses (Figure 7). In BC-1, high expression of *HIF-1α* led to increased ROS levels, a common characteristic of cancer-activated *HIF-1α* without upstream *NRF2* interference. On the other hand, LC-1 cells exhibited increases in both *NRF2* and *HIF-1α* expressions. *NRF2* upregulation in response to rising free radicals and ROS serves to maintain redox homeostasis and rescue cells from death, while *HIF-1α* induction is associated with cancer cell survival mechanisms under fluctuating intracellular ROS levels [50]. Hence, the observed inhibition of migration and invasion in BC-1 and LC-1 cells may not solely be attributed to intracellular ROS levels but likely involves other mechanisms or signaling molecules [51, 52].

The involvement of ROS in various cellular signaling transduction processes, including cancer cell growth, EMT, and metastasis, highlights its significance in cancer biology. However, it is important to acknowledge that cellular responses to ROS are highly diverse and depend on specific cell types. Further purification and investigation of phytochemicals, along with comprehensive cellular biology studies, are needed to unravel the intricate mechanisms underlying cancer cell migration, invasion, and ROS levels. These endeavors hold the potential to provide crucial insights into the complex interplay between ROS and cancer, thereby paving the way for the development of innovative therapeutic strategies targeting ROS-mediated processes in cancer.

## 5. Conclusions

The ethanolic extract of *C. inophyllum* demonstrated selective cytotoxicity, antiproliferation, and migration/invasion inhibition which was related to intracellular ROS reduction. The potent anticancer effects might be due to the availability of total phenolic content and total flavonoid content and their strong antioxidant properties. Notably, the prominent phytochemical component responsible for these antioxidant effects is 5-HMF. When tested on both PDCs and standard commercial cell lines of breast and lung cancers, the *C. inophyllum* extract exhibited specific antiproliferative effects that selectively targeted cancer cells. The extract's antioxidant properties efficiently attenuated intracellular ROS levels, consequently impeding the migration and invasion processes. This observation was further substantiated by gene expression analysis, revealing a significant increase in *E-cadherin* expression alongside notable decreases in *Twist-1*, *MMP-2*, *MMP-9*, *NRF2*, and *HIF-1α*. Therefore, the findings of this study suggest that the *C. inophyllum* extract could have the potential to be an alternative therapeutic agent for cancer treatment. Nevertheless, further research and development are still required to fully understand the mechanism of cancer therapy.

## Figures and Tables

**Table 1:** DPPH scavenging activity (IC_50_) of *C. inophyllum* extract.

Compounds	Linear equation	*R * ^2^	DPPH scavenging activity (IC_50_) *μ*g/ml
*C. inophyllum* extract	*y* = 0.3748*x* + 0.8484	0.9728	135.67 ± 0.24
Ascorbic acid	*y* = 0.8148*x* + 1.9497	0.9881	63.75 ± 0.13
Quercetin	*y* = 1.2539*x* + 1.1429	0.9943	38.96 ± 0.45

**Table 2:** Compounds identified in *C. inophyllum* extract by GC-MS.

No	RT	Compound	Class	Formula	M_W_	%
1	9.9863	Methyl 2-furoate	Furoic acid esters	C_5_H_4_O_3_	112.08	0.29
2	12.4181	2,3-Dihydro-3,5-dihydroxy-6-methyl-4H-pyran-4-one	Dihydropyranones	C_6_H_8_O_4_	114.12	2.96
3	14.5181	Pyrocatechol	Catechols	C_6_H_6_O_2_	110.11	1.37
4	15.5080	2,3-Dihydrobenzofuran	Benzofurans	C_8_H_6_O	118.13	0.76
5	15.9371	5-Hydroxymethylfurfural	Furans	C_6_H_6_O_3_	126.11	13.27
6	19.4161	2-Methoxy-4-vinylphenol	Phenols	C_9_H_10_O_2_	150.17	0.57
7	20.9610	Syringol	Phenols	C_8_H_10_O_3_	154.16	3.23
8	22.9522	2-Methoxyhydroquinone	Phenols	C_7_H_8_O_3_	140.14	1.15
9	31.3578	Antiarol	Phenols	C_9_H_12_O_4_	184.19	6.42
10	35.9755	Coniferyl alcohol	Phenols	C_10_H_12_O_3_	180.2	0.65
11	43.8947	Palmitic acid	Fatty acids	C_16_H_32_O_2_	256.42	0.22
12	45.0219	Ethyl palmitate	Fatty acid esters	C_18_H_36_O_2_	284.5	0.96
13	49.2561	8-Isocyano-6,7-dimethoxy-1-methylisoquinoline	Isoquinolines	C_13_H_12_N_2_O_2_	228.25	1.15
14	50.0115	Ethyl linoleate	Fatty acid esters	C_20_H_36_O_2_	308.5	0.60
15	50.2403	Ethyl elaidate	Fatty acid esters	C_20_H_38_O_2_	281.5	0.69
16	51.3447	3-Hydroxyxanthen-9-one	Xanthones	C_13_H_8_O_3_	212.2	0.62

**Table 3:** Basic information of cancer cells derived from patients.

Type of cancer	Age of patients	Gender	Diagnosis	Name of PDCs
Breast	54	Female	Invasive ductal carcinoma	BC-1
	54	Female	Invasive ductal carcinoma	BC-2
	50	Female	Invasive ductal carcinoma	BC-3

Lung	66	Female	Adenocarcinoma	LC-1
	79	Female	Adenocarcinoma	LC-2
	70	Male	Adenocarcinoma, moderately differentiated with focal squamous differentiation	LC-3

**Table 4:** The IC_50_ values of *C. inophyllum* extract and quercetin against PDCs of breast (BC-1, BC-2, and BC-3) and lung (LC-1, LC-2, and LC-3) cancers and in cancer cell lines of breast (MCF-7 and MDA-MB-231) and lung (A549 and NCI-H1299) cancers at 24 h and 72 h.

Cells	*C. inophyllum* extract		Quercetin	
	24 h (*μ*g/ml)	72 h (*μ*g/ml)	24 h (*μ*g/ml)	72 h (*μ*g/ml)
BC-1	>2,000	>2,000	282.88 ± 0.47	129.01 ± 0.46
BC-2	>2,000	>2,000	74.74 ± 0.11	57.92 ± 0.41
BC-3	>2,000	>2,000	368.49 ± 0.24	62.63 ± 0.23
LC-1	1.01 × 10^3^ ± 0.29	246.01 ± 0.21	305.18 ± 0.81	120.33 ± 0.31
LC-2	1.20 × 10^3^ ± 0.37	333.93 ± 0.83	203.43 ± 0.42	47.96 ± 0.77
LC-3	1.02 × 10^3^ ± 0.31	234.35 ± 0.46	38.52 ± 0.19	14.42 ± 0.36
MCF-7	404.78 ± 0.47	97.42 ± 0.65	1.75 × 10^3^ ± 0.25	195.44 ± 0.14
MDA-MB-231	>2,000	273.17 ± 0.18	1.48 × 10^3^ ± 0.35	64.21 ± 0.56
A549	1.14 × 10^3^ ± 0.12	144.64 ± 0.61	>2,000	1.17 × 10^3^ ± 0.83
NCI-H1299	1.38 × 10^3^ ± 0.64	183.86 ± 0.67	>2,000	110.41 ± 0.74
IMR-90	1.62 × 10^3^ ± 0.56	342.25 ± 0.44	377.04 ± 0.27	135.47 ± 0.64

**Table 5:** Selectivity index of *C. inophyllum* extract and quercetin against PDCs of breast (BC-1, BC-2, and BC-3) and lung (LC-1, LC-2, and LC-3) cancers and in cancer cell lines of breast (MCF-7 and MDA-MB-231) and lung (A549 and NCI-H1299) cancers at 24 h and 72 h.

Cells	*C. inophyllum* extract		Quercetin	
	24 h	72 h	24 h	72 h
BC-1	<1	<1	1.33	1.05
BC-2	<1	<1	5.04	2.34
BC-3	<1	<1	1.02	2.16
LC-1	1.60	1.39	1.24	1.13
LC-2	1.35	1.03	1.85	2.82
LC-3	1.59	1.46	9.79	9.39
MCF-7	4.00	3.51	<1	<1
MDA-MB-231	<1	1.25	<1	2.11
A549	1.42	2.37	<1	<1
NCI-H1299	1.17	1.86	<1	1.23

## Data Availability

The generated or analyzed data used to support the findings of this study are included within the article.
